# Dynamic Bus Travel Time Prediction Models on Road with Multiple Bus Routes

**DOI:** 10.1155/2015/432389

**Published:** 2015-07-30

**Authors:** Cong Bai, Zhong-Ren Peng, Qing-Chang Lu, Jian Sun

**Affiliations:** ^1^State Key Laboratory of Ocean Engineering, Shanghai Jiao Tong University, Shanghai 200240, China; ^2^School of Naval Architecture, Ocean and Civil Engineering, Shanghai Jiao Tong University, Shanghai 200240, China; ^3^Center for ITS and UAV Application Research, Shanghai Jiao Tong University, Shanghai 200240, China; ^4^Department of Urban and Regional Planning, University of Florida, Gainesville, FL 32611, USA

## Abstract

Accurate and real-time travel time information for buses can help passengers better plan their trips and minimize waiting times. A dynamic travel time prediction model for buses addressing the cases on road with multiple bus routes is proposed in this paper, based on support vector machines (SVMs) and Kalman filtering-based algorithm. In the proposed model, the well-trained SVM model predicts the baseline bus travel times from the historical bus trip data; the Kalman filtering-based dynamic algorithm can adjust bus travel times with the latest bus operation information and the estimated baseline travel times. The performance of the proposed dynamic model is validated with the real-world data on road with multiple bus routes in Shenzhen, China. The results show that the proposed dynamic model is feasible and applicable for bus travel time prediction and has the best prediction performance among all the five models proposed in the study in terms of prediction accuracy on road with multiple bus routes.

## 1. Introduction

Providing reliable and accurate bus travel and arrival times would be an effective way to improve the service of bus transit systems [1]. By using the advanced technologies such as automatic vehicle location (AVL) or automatic vehicle identification (AVI) systems or automatic passenger counters (APC), the level of service of traditional bus transit systems can be greatly improved. Generally speaking, the passengers are interested in the predicted travel times of the next buses and the predicted arrival times at the bus stop [2]. Therefore, the accuracy of the prediction results is very important for traditional bus transit systems.

In practice, particularly in cities like Shenzhen, China, it is very common to have several bus routes sharing the same road segments and bus stops. Passengers can choose different bus routes to reach their destinations. They would like to know when the next buses of multiple bus routes will arrive at the bus stop [3]. But few previous studies addressed the specific situation of multiple bus routes sharing the same road segments and bus stops to predict the bus travel times. This specific case is detailed in Section 3.1.

Three contributions have been made in this paper. First, a case that several bus routes share the same road segments and bus stops is addressed and detailed, which is very common in the transit-oriented cities like Shenzhen, Beijing, and Shanghai in China. Second, a dynamic bus travel time prediction model on road with multiple bus routes is developed using real-world data, which can fill the gap that there is no dynamic model for bus arrival time prediction focusing on the above case. It is expected that if the predicted arrival times of the next buses of multiple bus routes could be known by the passengers, it would save passengers' waiting times and decrease anxieties. The weighted average bus travel time of preceding buses of any route is considered as one of the input variables in the proposed models. Third, the performances of the dynamic models and the traditional models have been assessed and compared for forecasting bus arrival times on road with multiple bus routes. The performance comparison of different prediction models can provide valuable insight for researchers as well as practitioners.

The remainder of this paper is organized as follows: Section 2 reviews the related literature; Section 3 details the case of buses on road with multiple bus routes and provides the basic theory of the dynamic bus travel time prediction models, together with the input factors of the models; Section 4 presents a case study in Shenzhen, China, with the performance comparison of the five models; and Section 5 gives the conclusions and the suggestions for further study.

## 2. Literature Review

In the past decades, a variety of models and algorithms have been developed to predict bus arrival times or bus travel times. The most widely used ones can be classified into the following categories: historical average models, regression models, machine learning models including artificial neural network (ANN) models and support vector machines (SVMs) models, Kalman filtering-based models, and dynamic models.

### 2.1. Historical Average Models

Historical average models are based on the historic data and able to predicate the bus travel times or bus arrival times through previous bus trips. These models will be practical, useful, and reliable when the traffic flow is relatively small and stable. Jeong and Rilett [4] developed a historical model for predicting the link travel time between two bus stops, which was calculated as the average travel time between two bus stops minus the average dwell time at bus stops. Vanajakshi and Rilett [5] also suggested a historic approach in their study.

Historical average models could be valuable in the development of prediction models but the reliability of the prediction accuracy was limited.

### 2.2. Regression Models

Regression models use a multivariate statistical technique for examining the linear correlations between a set of independent variables and a single dependent variable [6]. Jeong and Rilett [4] proposed a set of multiple linear regression models to estimate travel times from current bus stop to the target bus stop. Distance, bus schedule adherence, and arrival time at one specific bus stop were chosen as the independent variables in regression models. Ramakrishna et al. [7] and Patnaik et al. [8] also established regression models in their studies with different independent variables.

Although different independent variables and different combinations of these independent variables were set in different regression models, the results suggested that the prediction performance of the regression models was good. In addition, multiple linear regression models have the ability to reveal the degree of importance of each independent variable.

### 2.3. Artificial Neural Network Models

Artificial neural network (ANN) models are very popular in forecasting bus travel times and bus arrival times. Chien et al. [9], Jeong and Rilett [4], Fan [10], Ramakrishna et al. [7], Kumar et al. [1], Yu et al. [11], and many other researchers had developed ANN models in their studies. Ramakrishna et al. [7] found that ANN model outperformed the regression model. Jeong and Rilett [4] suggested that ANN model outperformed the historical average model and the regression model in terms of prediction accuracy. Fan [10] drew the same conclusions as Jeong and Rilett [4].

Previous studies proved that ANN models had the ability to solve complex nonlinear relationships and they are very effective in bus travel time prediction.

### 2.4. Support Vector Machine Models

Recently, SVM had been proposed as a good technique for bus arrival time prediction and bus travel time prediction. There were many successful attempts in bus travel time prediction. Vanajakshi and Rilett [5] compared a number of different forecasting methods for travel time prediction including historic method, time series analysis, ANN, and SVM. Comparison showed that the performances of both SVM and ANN models were comparable to each other, and these two methods outperformed other methods. Yu et al. [12] developed a SVM-based model to predict the bus travel times of transit route number 23 in Dalian, China. The results showed that the SVM model outperformed the historic mean prediction model, the autoregressive integrated moving average, and the ANN model. Yu et al. [3] also compared four models, namely, SVM, ANN, *k*-nearest neighbors (*k*-NN) algorithm, and regression model. The results suggested that the performance of the SVM model was the best among the four models for bus arrival time prediction. Thissen et al. [13] and Wu et al. [14] also made contributions to the research of travel time prediction using SVM models.

SVM models proved to have better prediction performance than that of the ANN models. In general, SVM models outperformed other bus travel time prediction models in terms of prediction accuracy.

### 2.5. Kalman Filtering-Based Models

Kalman filtering algorithm was introduced by Chien and Kuchipudi [15] for travel time prediction because of its advantage in continuously updating the state variable as new observations. Chu et al. [16] developed a method for travel time estimation by applying the adaptive Kalman filter technique. This Kalman filter-based algorithm was tested in a stretch of freeway. Compared with the probe based method and the double detector based method, the proposed algorithm outperformed under both recurrent and nonrecurrent traffic conditions. In Yang's study [17], a discrete-time Kalman filter was used to predict arterial travel times. Although various approaches based on Kalman filter were explored to improve the prediction accuracy, this study lacks the comparison of other prediction models. Kumar [18] focused on a model-based Kalman filtering algorithm. Compared with a prediction method using space discretization, the proposed algorithm had better performance in prediction accuracy. In addition, in some other studies [11, 19–21], some travel time prediction models utilizing Kalman filter-based algorithm were developed.

There are many previous studies using Kalman filtering-based dynamic algorithm in travel time prediction. All these studies showed that Kalman filtering-based models are feasible and have a strong theoretical foundation in travel time prediction. However, most of these Kalman filtering-based models lack the performance comparison with other models and algorithms.

### 2.6. Dynamic Models

Different researchers have different opinions on dynamic models, and as a result different algorithms are proposed in the dynamic models.

Elhenawy et al. [22] developed a data clustering and genetic programming approach for modeling and predicting the dynamic travel times along freeways. Chen et al. [19] proposed a dynamic algorithm integrating the ANN model and Kalman filter-based algorithm, because the history data-based models had difficulty in dealing with dynamic traffic conditions. Results showed that this dynamic model was powerful in predicting bus arrival times along the service route. Yu et al. [11] proposed a hybrid model which was based on SVM and Kalman filtering technique to predict bus arrival times, which performed better than the ANN-based methods. Liu et al. [20] predicted urban arterial travel times with state space neural networks (SSNN) and Kalman filters. The Kalman filters algorithm was applied to train the SSNN model, which was different from that of Chen et al.'s [21] method. Chen et al. proposed an integrated bus rapid transit (BRT) vehicle travel time prediction model. This model used the SVM and Kalman filter algorithm to dynamically predict travel times. The Kalman filter algorithm was applied to adjust the bus travel times predicted by SVM. The prediction results of the proposed model outperformed the Kalman filter model, but it lacked the results comparison with that of SVM model. Besides, BRT vehicles (buses) operate on exclusive rights-of-way or bus lanes [23], which totally differs from the buses in the normal bus transit systems.

However, only Yu et al. [3] addressed the importance of buses on road with multiple bus routes. By integrating bus travel times of different bus routes on the same road segments, the estimation accuracy of traffic conditions could be improved. The limitation of Yu's research is that no dynamic model was introduced and only peak hours were studied. It needs further study in this specific case of buses sharing the same road segments and bus stops.

In summary, previous researches have been conducted on the research field of bus travel/arrival time prediction for a single bus route, but few researches addressed the case of buses on road with multiple bus routes, which is worth further research in order to improve the prediction accuracy. The previous researches only used the information of the same bus route to predict the bus travel/arrival times, but the integration of bus information of multiple bus routes was not included in these studies. Although Yu et al. [3] made some contributions to this problem, no dynamic model was developed in the study and whether a dynamic model could further improve the prediction accuracy remained unknown. Since studies in recent years proved that SVM and ANN models outperformed other models in prediction accuracy, in this study five models, including pure ANN, pure SVM, pure Kalman, ANN-Kalman (ANN-Kalman model refers to the model based on ANN and Kalman filtering-based algorithm), and SVM-Kalman (SVM-Kalman model is short for the model based on SVM and Kalman filtering-based algorithm) models, are proposed for bus travel time prediction on road with multiple bus routes.

## 3. Problem Descriptions and Model Developments

The dynamic model consists of two main components: the first component is the support vector machines (SVMs) model estimating the baseline bus travel times on road with multiple bus routes; the second component is the Kalman filtering-based dynamic algorithm, so the prediction results of the first component can be adjusted based on the latest travel time information.

### 3.1. Problem Descriptions

In transit-oriented cities like Shenzhen, China, it is very common that several bus routes share the same road segments and bus stops.

**Figure 1:**
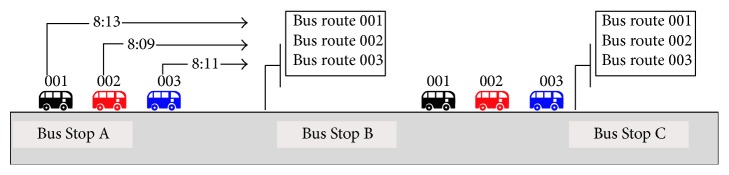
Example of multiple bus routes sharing the same road segments.

Figure 1 shows the example of multiple bus routes sharing the same road segments. For example, a passenger expects to travel from Bus Stop B to Bus Stop C where there are three bus routes, namely, bus route 001, bus route 002, and bus route 003. Therefore, this passenger can choose any bus of these three bus routes to reach Bus Stop C. Assuming that the predicted arrival times of the next buses of all three routes at Bus Stop B (e.g., 8:13, 8:09, and 8:11, resp.) can be known by this passenger, he or she will wait for the next bus of bus route 002, which will save the passenger' waiting time at Bus Stop B.

Generally speaking, the operating characteristics and influencing factors for buses operating on road with multiple bus routes are different from those with single bus route. Bus travel time on road with single bus route is mainly affected by the traffic conditions on road, but bus travel time on road with multiple bus routes could also be affected by the buses of other bus routes as well as the traffic conditions. Due to the limited capacity of bus stops, the buses might queue up at the bus stops and therefore the bus travel time becomes much longer and unpredictable. Thus, to find a feasible and applicable predicting method for bus arrival times on road with multiple bus routes is very meaningful and important.

### 3.2. Support Vector Machine Model

In this section, the basic idea of SVM is briefly introduced. SVM model can map the training data from the input space into a higher dimensional feature space. In this higher dimensional feature space, a separating hyper plane is constructed which can make the maximum margin in the feature space. Points on the edge are called the support vectors.

Let a set of points (*x*_1_, *y*_1_), (*x*_2_, *y*_2_),…, (*x*_*k*_, *y*_*k*_) be a *n*-dimensional vector. Each *x*_*i*_ denotes the input space of the sample, which has the corresponding output value *y*_*i*_.

The SVM function has the following formula:

(1) $$\begin{array}{c} f\left(\;x\;\right)=\omega \emptyset \left(\;x\;\right)+b, \end{array}$$

where the function *∅*(*x*) can relate the input space to a higher dimensional feature space.

The SVM aims to find the best separating hyper plane which is defined to minimize the following cost function *Q*. The values of *ω* and *b* are also determined by minimizing the cost function *Q*:

(2) $$\begin{array}{c} Q=\frac{1}{2}{\left\|\;\omega \;\right\|}^{2}+C\frac{1}{k}\overset{k}{\underset{i=1}{\sum }}L_{\varepsilon }\left(\;y_{i},f\left(\;x_{i}\;\right)\;\right), \end{array}$$

where *C* is a constant which evaluates the trade-off between the empirical risk and the smoothness of the model. The vector *ω* can be expressed by the data points:

(3) $$\begin{array}{c} \omega =\overset{k}{\underset{i=1}{\sum }}\left(\;a_{i}-a_{i}^{*}\;\right)\emptyset \left(\;x_{i}\;\right), \end{array}$$

where *a*_*i*_ and *a*_*i*_^*∗*^ are Lagrange multipliers.

By introducing (3) to (1), (1) can be written as follows:

(4) $$\begin{array}{c} f\left(\;x\;\right)=\overset{k}{\underset{i=1}{\sum }}\left(\;a_{i}-a_{i}^{*}\;\right)\emptyset \left(\;x_{i}\;\right)\cdot \emptyset \left(\;x\;\right)+b. \end{array}$$

Some common kernel functions are shown in Table 1.

In previous studies [3, 5, 11, 13], normally the RBF kernel is used for regression. By introducing RBF kernel function into (4), it can be rewritten as

(5) $$\begin{array}{c} f\left(\;x\;\right)=\overset{k}{\underset{i=1}{\sum }}\left(\;a_{i}-a_{i}^{*}\;\right)K\left(\;x_{i},x_{j}\;\right)+b. \end{array}$$

Since the RBF kernel is selected, the key problem identifies the best combination of parameter *C* and parameter *γ*. *C* defines the cost of the penalty that determines the penalties to estimation errors; *γ* represents radius that determines the data inside the tube to be ignored in regression [14]. Different combination of parameter *C* and parameter *γ* has a significant impact on the prediction accuracy; therefore it is necessary to optimize the combination of parameter *C* and parameter *γ*. There are several common techniques to obtain the best combination of parameters *C* and *γ*, including cross validation (CV), genetic algorithm (GA), and particle swarm optimization (PSO). All the above three methods are introduced in this study to get most optimal combination of parameter *C* and parameter *γ*.

### 3.3. Kalman Filtering-Based Dynamic Algorithm

Although the performances of SVM or ANN models outperform other models in terms of prediction accuracy, the SVM or ANN models still cannot adjust the prediction results dynamically. The SVM or ANN model is based on the historical data, and no matter how they are trained and tested they can only estimate the bus travel times based on historical data but not the real-time information. So the Kalman filtering-based dynamic algorithm is proposed in this dynamic model so as to take full use of the latest bus travel time data.

Let *x*_*k*_ denote the bus travel time at current time step *k* that needs to be predicted, *A*_*k*−1_ denotes the state transition parameter relating *x*_*k*−1_ to *x*_*k*_, and *w*_*k*−1_ denotes the process noise term that has a normal distribution with zero mean and a variance of *Q*_*k*−1_. Then the state equation can be expressed as

(6) $$\begin{array}{c} x_{k}=A_{k-1}x_{k-1}+w_{k-1}. \end{array}$$

Let *y*_*k*_ denote the measured state at current time step *k*, *H*_*k*_ denotes the observation matrix, and *v*_*k*_ denotes the measurement noise term that has a normal distribution with zero mean and a variance of *R*_*k*−1_. *w*_*k*−1_ and *v*_*k*_ are assumed to be independent of each other. Thus the measurement equation can be written as follows:

(7) $$\begin{array}{c} y_{k}=H_{k}x_{k}+v_{k}. \end{array}$$

The state transition parameter *A*_*k*−1_ can be calculated by the data in the previous time step.

Only the data of travel time is considered in this study and both *x*_*k*_ and *y*_*k*_ denote one-dimensional variable, so *A*_*k*−1_ = (1) and *H*_*k*_ = (1). The state *x*_*k*_ should follow

(8) $$\begin{array}{c} x_{k}=x_{k-1}+w_{k-1} \end{array}$$

with a measurement of *y*_*k*_:

(9) $$\begin{array}{c} y_{k}=x_{k}+v_{k}. \end{array}$$

Then the filtering procedure is shown as follows [17, 24].

**Step 1 (initialization).**

Set *t* = 0 and let $E\left[x_{0}\right]={\hat{x}}_{0}$ and $E\left\{{\left[x_{0}-{\hat{x}}_{0}\right]}^{2}\right\}=P_{0}$ and *E*[*w*(*i*)*v*(*j*)] = 0 for all *i*, *j*, where ${\hat{x}}_{0}$ is the predicted bus travel time at time step 0 and *P*_*k*_ denote the covariance of the estimation error at time step *k*.

**Step 2 (extrapolation).**

Consider the following:

 Extrapolate state estimate:
  (10) $$\begin{array}{c} {\hat{x}}_{k}^{-}=A_{k-1}{\hat{x}}_{k-1}^{}. \end{array}$$
 Extrapolate error covariance:
  (11) $$\begin{array}{c} P_{k}^{-}=A_{k-1}P_{k-1}^{}{A_{k-1}}^{T}+Q_{k-1}, \end{array}$$
 where ${\hat{x}}_{k}^{-}$ denotes the prior estimate; the hat “$\hat{\;\;\;\;}$” means that it is an estimated value and the superscript “−” is a reminder meaning that this estimated value is the best estimated value.

**Step 3 (Kalman gain calculation).**

Consider the following

(12) $$\begin{array}{c} K_{k}=P_{k}^{-}{\left(\;P_{k}^{-}+R_{k}\;\right)}^{-1}, \end{array}$$

where *K*_*k*_ is the blending factor, and the optimal estimation problem is to find a particular *K*_*k*_ to minimize the performance criterion.

**Step 4 (update).**

Consider the following.

 Update state estimate:
  (13) $$\begin{array}{c} {\hat{x}}_{k}={\hat{x}}_{k}^{-}+K_{k}\left(\;y_{k}-{\hat{x}}_{k}^{-}\;\right). \end{array}$$
 Update error covariance:
  (14) $$\begin{array}{c} P_{k}=\left(\;I-K_{k}\;\right)P_{k}^{-}. \end{array}$$

**Step 5 (next iteration).**

Let *k* = *k* + 1 and go back to Step 2 until the circulation is finished.

Detailed derivations of Kalman filtering equations can be found elsewhere [25].

### 3.4. Dynamic Model

**Figure 2:**
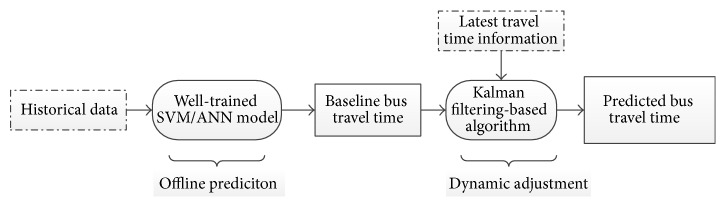
Framework of the dynamic model.

Figure 2 depicts the framework of the dynamic model. The framework consists of two steps, namely, the offline prediction step and the dynamic adjustment step. The first step is the offline prediction, which uses the historical bus travel time data and the well-trained SVM or ANN models. The output of the first step is the baseline bus travel time, which serves as the input of the second step. The second step is the dynamic adjustment. In the second step, the Kalman filtering-based algorithm can adjust the baseline bus travel time with the latest travel time data. This dynamic model is SVM-Kalman model.

### 3.5. Model Inputs

The inputs considered in the proposed models include the following factors.

*(1) Time of Day*. At different time of day the bus travel times are different. Especially at morning and afternoon peak hours, the bus travel times will increase significantly. Thus, the factor time of day should be considered as an input of the models, which is expressed as time of day.

*(2) Road Segment*. Different road segments have different number of intersections (signalized or unsignalized), road segment length, traffic conditions, and traffic flow composition. All these differences can result in the changes of bus travel times. Thus, road segment should be a factor in the models, which is expressed as segment.

*(3) The Weighted Average Bus Travel Time of Preceding Buses of Any Route*. In order to simplify the statement, the term “the preceding bus(es)” refers to the last bus(es) that has(ve) just traveled along the road segment with multiple bus routes.

The travel time of the last preceding bus has more contribution to the weighted average bus travel time than that of other further buses. A simple weighted method is taken into consideration in order to weight travel time of each preceding bus, which is the inverse of the time headway between the preceding buses and the bus for prediction at the beginning bus stop on a road segment:

(15) t−L=∑j=1m1/TjΓTL,j,Γ=∑j=1m1Tj,

where *L* denotes the set of bus routes along the same segment; *T*_*L*,*j*_ is the bus travel time in road segment of the *j*th preceding bus; *T*_*j*_ is the time headway between the preceding buses and the bus for travel time prediction at the beginning bus stop of the road segment; and ${\overset{-}{t}}_{L}$ is the weighted average bus travel time of several preceding buses of any routes among bus routes set *L*.

According to Yu et al. [3], only 3 preceding buses are considered in this study; namely, *m* = 3.

*(4) The Bus Travel Time of the Preceding Bus on the Same Bus Route*. Similar information of bus operation can be provided by the buses of the same bus route, so the bus travel time of the preceding bus of the same bus route is considered, which is denoted by *t*_*l*_.

Thus, the prediction of bus travel time *t*_predicted_ on road with multiple bus routes can be formulated as follows:

(16) $$\begin{array}{c} t_{\text{predicted}}=f\left(\;\text{time of day},\text{segment},{\overset{-}{t}}_{L},t_{l}\;\right). \end{array}$$

## 4. Case Study

### 4.1. Model Performance Measures

The prediction results are evaluated in terms of prediction accuracy by the following three measures: the mean absolute error (MAE), the mean absolute percentage error (MAPE), and the root mean square error (RMSE). Each measure is calculated as follows:

(17) MAE=∑tobserved−tpredictedN,MAPE=1N∑tobserved−tpredictedtobserved,RMSE=∑tobserved−tpredicted2N−1,

where *t*_observed_ is the observed bus travel time; *t*_predicted_ is the predicted bus travel time; and *N* is the number of the bus trips observed.

### 4.2. Study Bus Routes and Data Collection

The proposed five models for bus travel time prediction have been evaluated by the real-world data in Shenzhen, China. In Shenzhen, the buses have been equipped with the devices that can record the real-time information, including position information, the arrival times, and the departure times at bus stops. All the data are transferred to the Transport Commission of Shenzhen Municipality in real-time.

**Figure 3:**
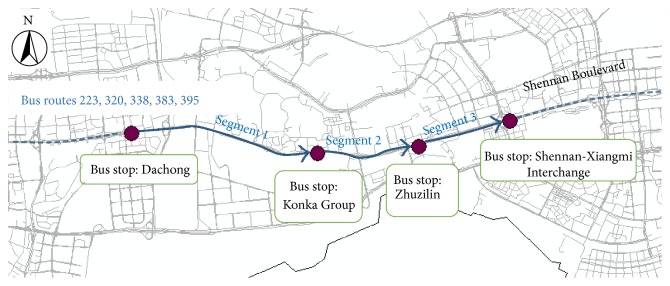
Studied road segments with multiple bus routes.

There are five bus routes on the road segment from Bus Stop Dachong to Bus Stop Shennan-Xiangmi Interchange along the Shennan Boulevard. Thus, this road segment is selected to test the proposed models in this study, which is illustrated in Figure 3.

From Bus Stop Dachong to Bus Stop Shennan-Xiangmi Interchange, there are five bus routes, which are bus route 223, bus route 320, bus route 338, bus route 383, and bus route 395. These four bus stops constitute three road segments, namely, segment 1 (from Bus Stop Dachong to Bus Stop Konka Group), segment 2 (from Bus Stop Konka Group to Bus Stop Zhuzilin), and segment 3 (from Bus Stop Zhuzilin to Bus Stop Shennan-Xiangmi Interchange).

The data collection was carried out from October 12, 2014, to October 25, 2014, in weekdays during the bus operation time (06:00 a.m.–23:00 p.m.). Bus arrival times, departure times, and license plate numbers are recorded at each bus stop.

After data filtering, Table 2 shows the sample size and descriptive statistics of valid observations during the 2 weeks.

For both the input and output data sets, to avoid numerical difficulties during the calculation, the data sets are scaled to the range of 0 and 1 before modeling. The calculation formula is as follows:

(18) $$\begin{array}{c} x_{i}^{\prime }=\frac{x_{i}-\min\left(x_{i}\right)}{\max\left(x_{i}\right)-\min\left(x_{i}\right)}, \end{array}$$

where *x*_*i*_ denotes the *i*th value of the input or output data set *X* = {*x*_1_, *x*_2_, *x*_3_,…, *x*_*n*_}; min⁡(*x*_*i*_) denotes the minimum value of the data set *X*; and max⁡(*x*_*i*_) denotes the maximum value of the data set *X*.

### 4.3. Model Identifications

All the data are divided into two parts, namely, the training data set and testing data set. Both ANN model and SVM model are trained and tested with the same data sets. The bus travel time observations on October 16, 2014, and October 23, 2014, are set as testing data set, and other observations are set as the training data set. 

*(1) SVM Model*. Three methods including cross validation (CV), genetic algorithm (GA), and particle swarm optimization (PSO) are tested to identify the best combination of parameter *C* and parameter *γ*. According to the model performance measures in Section 4.1, PSO method outperforms the other two methods since it has the smallest values of MAPE, RMSE, and MAE. The best combination of parameters is *C* = 0.1 and *γ* = 4.28575. 

*(2) ANN Model*. In order to evaluate the performance of the proposed dynamic model, an ANN-Kalman model is constructed using the same data sets as the SVM-Kalman model.

ANN model is a mathematical model simulating the neural structure of the human brain, which is suitable to model relationships that are difficult to explain or very complex between the inputs and outputs. ANN model requires two phases, the training phase and the testing phase.

The network architecture of ANN model in this study has three layers, which are an input layer, a hidden layer, and an output layer. During the training phase, the most commonly used algorithm is the back-propagation algorithm. The back propagation algorithm and the hyperbolic tangent sigmoid transfer function are used in this study. Different number of neurons in the hidden layer is tested in the back-propagation neural network model in order to identify the suitable well-trained one.

The final ANN architecture consists of the same input features as the SVM model, six neurons in the hidden layer, and one neuron in the output layer.

### 4.4. Results and Discussion

The performances of the five models, namely, SVM-Kalman, ANN-Kalman, SVM, ANN, and Kalman, for the three road segments are presented in Table 3.

From Table 3, the SVM-Kalman model and ANN-Kalman model outperform the pure ANN, SVM, and Kalman models, in terms of MAE, MAPE, and RMSE. It indicates that the proposed dynamic models can improve the prediction accuracy significantly and ensure better forecasting results of bus travel times.

In addition, SVM-Kalman model has slightly better prediction performance than that of the ANN-Kalman model. On road segment 1, the SVM-Kalman model outperforms the ANN-Kalman model by 0.14 of MAE, 0.01 of MAPE, and 0.13 of RMSE and on road segment 3 by 1.11 of MAE, 0.64 of MAPE, and 1.74 of RMSE. On road segment 2, the ANN-Kalman model outperforms the SVM-Kalman model by 0.12 of RMSE, but the SVM-Kalman model outperforms the ANN-Kalman model by 0.01 of MAE and 0.14 by MAPE as well.

The prediction performance of SVM-Kalman model is much better than that of pure SVM, ANN, and Kalman models. Taking road segment 1 as an example, compared with the pure SVM model, the MAE of the SVM-Kalman model reduces from 52.70 s to 22.66 s, MAPE reduces from 10.24% to 4.33%, and RMSE reduces from 74.89 s to 30.17 s. The values of MAE, MAPE, and RMSE of the SVM-Kalman model also drop a lot compared with the pure ANN and Kalman models. Similarly, the same downward trend of MAE, MAPE, and RMSE happens on road segment 2 and road segment 3.

In summary, based on the results of case study, the performance of the SVM-Kalman model for bus travel time prediction on road with multiple bus routes is feasible. In general, the SVM-Kalman model slightly outperforms the ANN-Kalman model; the SVM-Kalman model outperforms the pure SVM, ANN, and Kalman models a lot.

## 5. Conclusions 

This paper investigated the dynamic travel time prediction models for buses on road with multiple bus routes. The weighted average bus travel time of preceding buses of any route was introduced as one of the input variables in the proposed five models, namely, pure ANN model, pure SVM model, pure Kalman model, ANN-Kalman model, and SVM-Kalman model. The detailed theories of the support vector machine and Kalman filtering-based dynamic algorithm were presented in this paper, together with the structure of the dynamic bus travel time prediction models on road with multiple bus routes. To evaluate the proposed model, bus travel time data were collected by the devices equipped on the buses in Shenzhen, China, for two weeks during weekdays from Bus Stop Dachong to Bus Stop Shennan-Xiangmi Interchange on the Shennan Boulevard. The results showed that the proposed dynamic models outperformed the traditional pure SVM, ANN, and Kalman models. Furthermore, the comparison results showed that in general the SVM-Kalman model was the most accurate one among all the models. The SVM-Kalman model was a little better than the ANN-Kalman model in terms of prediction accuracy, but it outperformed the pure SVM, ANN, and Kalman models.

In this paper, only the data of the eastbound direction was collected for model comparison. Further studies are suggested to collect much more data, and more factors such as the weather condition and the travel times of other type vehicles should be considered in the models.

## Figures and Tables

**Table 1:** Common kernel functions.

Kernel	Function
Linear kernel	*K*(*x*_*i*_, *x*_*j*_) = *x*_*i*_ · *x*_*j*_
Polynomial kernel	*K*(*x*_*i*_, *x*_*j*_) = (*x*_*i*_ · *x*_*j*_ + 1)^*d*^
RBF kernel	*K*(*x*_*i*_, *x*_*j*_) = exp⁡(−*γ*‖*x*_*i*_, *x*_*j*_‖^2^)⁡
Sigmoid kernel	*K*(*x*_*i*_, *x*_*j*_) = tanh⁡(*b*(*x*_*i*_ · *x*_*j*_) + *c*)⁡

**Table 2:** Sample size of each route and descriptive statistics.

Bus route number	Road segment number	Sample sizes	Descriptive statistics			
			Min (s)	Max (s)	Average (s)	SD^*∗*^
223	Segment 1	1628	328	828	523.85	79.88
	Segment 2	1502	129	545	248.96	57.26
	Segment 3	1394	150	447	207.38	28.93

320	Segment 1	1086	345	766	518.15	67.62
	Segment 2	1162	175	485	280.98	52.22
	Segment 3	1146	102	555	174.53	28.11

338	Segment 1	1420	328	813	540.05	71.93
	Segment 2	1434	173	554	311.15	53.41
	Segment 3	1456	114	601	227.24	37.09

383	Segment 1	1436	194	818	506.88	81.83
	Segment 2	1316	124	495	262.81	56.05
	Segment 3	1190	113	424	183.15	35.07

395	Segment 1	1558	231	823	514.63	70.14
	Segment 2	1568	179	674	321.06	68.6
	Segment 3	1520	104	560	228.44	38.44

^*∗*^SD means standard deviation.

**Table 3:** Comparison of prediction errors for five models^*∗*^.

	Road segment 1			Road segment 2			Road segment 3		
	MAE	MAPE	RMSE	MAE	MAPE	RMSE	MAE	MAPE	RMSE
ANN	55.55	10.52	74.89	49.31	17.60	63.14	25.64	12.70	33.25
SVM	53.70	10.24	71.43	47.33	16.73	60.55	24.79	11.91	32.65
Kalman	54.33	10.68	75.39	48.82	17.98	60.59	25.68	13.06	32.97
ANN-Kalman	22.72	4.34	30.30	19.45	6.96	25.25	10.51	5.10	14.34
SVM-Kalman	22.66	4.33	30.17	19.44	6.82	25.37	9.40	4.46	12.60

^*∗*^MAE and RMSE are in units of second (s) and MAPE is in units of percentage (%).

## References

[1] Kumar V., Kumar B. A., Vanajakshi L. Comparison of model based and machine learning approaches for bus arrival time prediction.

[2] Ma C. Q., Wang Y. P., Chen K. M. (2007). Competition model between urban rail and bus transit. *Journal of Transportation Systems Engineering and Information Technology*.

[3] Yu B., Lam W. H. K., Tam M. L. (2011). Bus arrival time prediction at bus stop with multiple routes. *Transportation Research Part C: Emerging Technologies*.

[4] Jeong R., Rilett L. R. Bus arrival time prediction using artificial neural network model.

[5] Vanajakshi L., Rilett L. Support vector machine technique for the short term prediction of travel time.

[6] Marx M. L., Larsen R. J. (2006). *Introduction to Mathematical Statistics and Its Applications*.

[7] Ramakrishna Y., Ramakrishna P., Lakshmanan V. Bus Travel Time Prediction Using Global Positioning System Data[EB/OL]. http://www.gisdevelopment.net/proceedings/mapindia/2006/student%20oral/mi06stu_84.htm/.

[8] Patnaik J., Chien S., Bladikas A. (2004). Estimation of bus arrival times using APC data. *Journal of Public Transportation*.

[9] Chien S. I.-J., Ding Y., Wei C. (2002). Dynamic bus arrival time prediction with artificial neural networks. *Journal of Transportation Engineering*.

[10] Fan W. D. (2014). Artificial neural network travel time prediction model for buses using only global positioning system data. *Journal of Public Transportation*.

[11] Yu B., Yang Z.-Z., Chen K., Yu B. (2010). Hybrid model for prediction of bus arrival times at next station. *Journal of Advanced Transportation*.

[12] Yu B., Yang Z. Z., Wang J. (2010). Bus travel-time prediction based on bus speed. *Proceedings of the Institution of Civil Engineers: Transport*.

[13] Thissen U., Van Brakel R., De Weijer A. P., Melssen W. J., Buydens L. M. C. (2003). Using support vector machines for time series prediction. *Chemometrics and Intelligent Laboratory Systems*.

[14] Wu C.-H., Ho J.-M., Lee D. T. (2004). Travel-time prediction with support vector regression. *IEEE Transactions on Intelligent Transportation Systems*.

[15] Chien S. I.-J., Kuchipudi C. M. (2003). Dynamic travel time prediction with real-time and historic data. *Journal of Transportation Engineering*.

[16] Chu L., Oh S., Recker W. Adaptive Kalman filter based freeway travel time estimation.

[17] Yang J.-S. Travel time prediction using the GPS test vehicle and Kalman filtering techniques.

[18] Kumar B. A. (2013). *Pattern-Based Bus Travel Time Prediction under Heterogeneous Traffic Conditions*.

[19] Chen M., Liu X., Xia J., Chien S. I. (2004). A dynamic bus-arrival time prediction model based on APC data. *Computer-Aided Civil and Infrastructure Engineering*.

[20] Liu H., van Zuylen H., van Lint H., Salomons M. (2006). Predicting urban arterial travel time with state-space neural networks and Kalman filters. *Transportation Research Record*.

[21] Chen X.-M., Gong H.-B., Wang J.-N. (2012). BRT vehicle travel time prediction based on SVM and Kalman filter. *Journal of Transportation Systems Engineering and Information Technology*.

[22] Elhenawy M., Chen H., Rakha H. A. (2014). Dynamic travel time prediction using data clustering and genetic programming. *Transportation Research Part C: Emerging Technologies*.

[23] Zimmerman S., Clinger J., Rutherford S. (2003). *Bus Rapid Transit*.

[24] Huang S. H., Ran B. (2003). *An Application of Neural Network on Traffic Speed Prediction under Adverse Weather Condition*.

[25] Kalman R. E. (1960). A new approach to linear filtering and prediction problems. *Journal of Basic Engineering*.

